# Latest Trends in Biosensing for Microphysiological Organs-on-a-Chip and Body-on-a-Chip Systems

**DOI:** 10.3390/bios9030110

**Published:** 2019-09-19

**Authors:** Sebastian Rudi Adam Kratz, Gregor Höll, Patrick Schuller, Peter Ertl, Mario Rothbauer

**Affiliations:** Institute of Applied Synthetic Chemistry and Institute of Chemical Technologies and Analytics, Faculty of Technical Chemistry, Vienna University of Technology, Getreidemarkt 9, 1060 Vienna, Austria; sebastian.kratz@tuwien.ac.at (S.R.A.K.); gregor.hoell@tuwien.ac.at (G.H.); patrick.schuller@tuwien.ac.at (P.S.); peter.ertl@tuwien.ac.at (P.E.)

**Keywords:** organ-on-a-chip, microphysiological systems, body-on-a-chip, biosensors, optical biosensors, electrical biosensors

## Abstract

Organs-on-chips are considered next generation *in vitro* tools capable of recreating *in vivo* like, physiological-relevant microenvironments needed to cultivate 3D tissue-engineered constructs (e.g., hydrogel-based organoids and spheroids) as well as tissue barriers. These microphysiological systems are ideally suited to (a) reduce animal testing by generating human organ models, (b) facilitate drug development and (c) perform personalized medicine by integrating patient-derived cells and patient-derived induced pluripotent stem cells (iPSCs) into microfluidic devices. An important aspect of any diagnostic device and cell analysis platform, however, is the integration and application of a variety of sensing strategies to provide reliable, high-content information on the health status of the *in vitro* model of choice. To overcome the analytical limitations of organs-on-a-chip systems a variety of biosensors have been integrated to provide continuous data on organ-specific reactions and dynamic tissue responses. Here, we review the latest trends in biosensors fit for monitoring human physiology in organs-on-a-chip systems including optical and electrochemical biosensors.

## 1. Introduction

After decades of progress in biomedical research, tissue engineering and pharmacology including combinatorial biochemical approaches and therapeutic strategies overall drug failure rates remain unchanged and regulatory approval rates for new drugs and therapies are still declining. It is well known that state-of-the-art *in vivo* animal tests often fail to predict human outcome and possible side effects during clinical trials [1,2,3], which has resulted in a renewed effort to develop more predictable and physiological relevant *in vitro* human three-dimensional (3D) tissue models. Among others, the establishment of microphysiological systems, often also referred to as “organ-on-a-chip technology”, is considered an emerging technology capable of engineering complex 3D tissue models exhibiting organ-like physiology or pathophysiological tissue level responses [4]. In these miniaturized systems the biophysical and biochemical microenvironment accounting for the respective native tissue architecture has been recapitulated for a range of organs including lung [5], liver [6], gut [7], kidney [8], blood-brain-barrier [9], vasculature [10,11], heart [12,13], skeletal muscle [14], placenta [15], as well as neuronal systems [16]. While cell barrier models are predominantly used in combination with two-dimensional (2D) polymeric or proteinaceous porous membranes to investigate the physiology, integrity and function of human cell barriers [17], 3D scaffolds including natural and synthetic hydrogels and polymers or scaffold-free cellular self-assembly strategies are exploited to generate tissue-level architecture [18,19]. More recently, organs-on-a-chip systems incorporate biophysical force to stimulate and trigger physiological phenotypes and functions [9,20,21]. A range of biomechanical forces including shear, strain, stretch and compression have been applied using either integrated mechanical actuators and flexible membranes or elevated fluid flow [22,23,24,25,26]. It is important to note that fluid mechanical forces can also influence material uptake and toxicology [27], which needs to be considered and evaluated during on-chip cell culture handling. The most prominent examples of recreating organotypic biomechanics involve a breathing lung and moving gut tissue using lateral vacuum to exert strain on a thin flexible porous membrane and in turn stimulate epithelial cell barriers to be tighter and more *in vivo* like [5,28]. In addition, control over spatio-temporal biochemical cues is a hallmark of any microfluidic cell-based 3D system due to the natural establishment of gradients of growth factors, waste products and oxygen supply within the 3D cell model [11,29]. In other words, 3D cell cultures can experience not only constant media and growth factor supply as well as waste removal but are also exposed to internal cues such as hypoxia, growth factor secretion, cell-cell interaction etc. Another major benefit of microfluidic technology is that several protocols for rapid prototyping and function integration of microdevices are available that allow fast and inexpensive design and fabrication of custom-made integrated platforms based on industrial polymers [30,31,32,33]. This inherent design flexibility of microfluidic devices including size, geometry, biointerface, flow conditions and integration of microfluidic components such as mixer, actuators, heaters and micropumps as well as embedded microsensors have severely advanced the performance of organs-on-a-chip systems by providing controlled near-native biochemical environments and reproducible measurement conditions [34]. Aside from obvious microscopy and off-chip analysis schemes, a range of electrical, magnetic and optical biosensing approaches have been reported over the years for monitoring of cell populations within microfabricated systems [35,36,37]. Despite recent advances, organs-on-a-chip systems predominantly rely on microscopical and off-chip analysis techniques for data acquisition, due to familiarity of cell biologist with cell-culture stains and ELISA technology.

To provide an overview on available and potential biosensing strategies, this review addresses recent advances in embedded microsensors in organ-on-a-chip devices, thus providing an outlook on future trends in organ-on-a-chip technology. Literature for this review was selected based on the following selection criteria: (i) up to date (< 3 years), (ii) organ- or microphysiolgical model excluding reports on cells placed on top of a biosensor, (iii) no microscopic/fluorescent imaging, (iv) no off-chip analysis, and (v) biosensor must be part of the microdevice platform. It has to be noted that aside from the classical definition of the term “biosensor”, this review also mentions cell-based microsensors that use a living cell as biorecognition element.

## 2. Biosensors for Organs-on-a-Chip Systems with Single Tissue Models

In the following section integrated biosensors are reviewed that have been applied for single tissue or organ type/function including metabolism, barrier models, muscular systems and neuronal models.

### 2.1. Biosensors for Analysis of Organ & Cancer Tissue Metabolism

**Figure 1:**
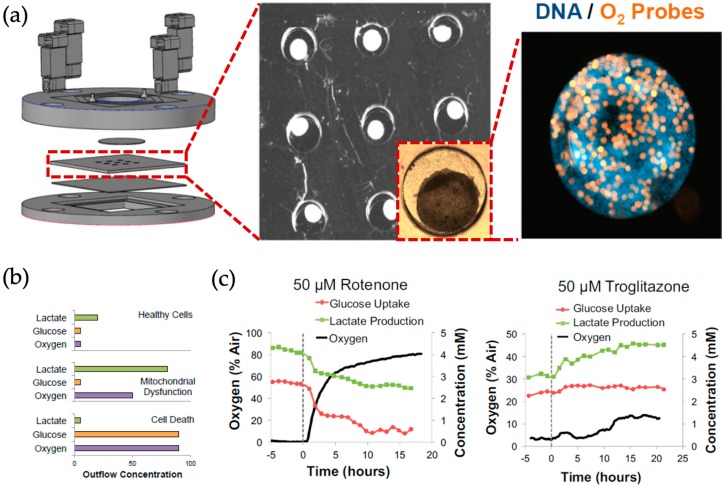
(**a**) Liver-on-a-chip for multiplexed culture of nine HepG2/C3A liver organoids with organoid integrated 400 µm oxygen sensing microprobes. (**b**) Correlation of oxygen uptake, glucose uptake and lactate production in healthy cells, dead cells and cells with mitochondrial dysfunction. (**c**) Influence of rotenone and troglitazone exposure on oxygen uptake, glucose uptake and lactate production of HepG2/C3A organoids. [41] Copyright 2016 National Academy of Science.

Non-invasive analysis of cell metabolism in organs-on-a-chips can be used to gain information on the health status of the tissue model. In order to analyze metabolism often small molecules, e.g., oxygen or glucose, are measured, thus providing additional information to standard end-point assays such as ELISA and PCR [38]. Especially in cancer research, information of cancer cell metabolism can shed light on tumor development and help to evaluate the efficacy of anti-cancer drugs [39]. For instance, Perrier et al. presented an automated microfluidic system containing MEA technology for on-line and real-time monitoring of glucose-dependent electrical activity of pancreatic islets. The described non-invasive sensing detected slow membrane potential shifts reflecting glucose concentration-dependent (3–15 mM) micro-organ activation. This setup was used to automatically identify and rank small increases of glucose levels in real time exhibiting a sensor response time of 40 µs (e.g., physiological glucose range). The authors envision this approach to facilitate monitoring of islets-on-chip systems in diabetes disease models, as well as performing maturation quality control during the production of stem-cell derived pancreatic islets [40]. Additionally, Bavli et al. reported a device capable of maintaining and monitoring liver organoids (consisting of HepG2/C3A cells) on-chip for a period of 28 days (Figure 1) using immobilized phosphorescence particles containing an oxygen sensitive ruthenium dye [41]. Additionally, embedded electrochemical glucose and lactate sensors based on platinum electrodes were used to assess the impact of various drugs on mitochondrial function and cell viability. Since even minute metabolic changes in the presence of drugs can be detected, real-time monitoring of mitochondrial dysfunction can serve as sensitive indicator of chemical toxicity even before cell necrosis or apoptosis occurs.

**Figure 2:**
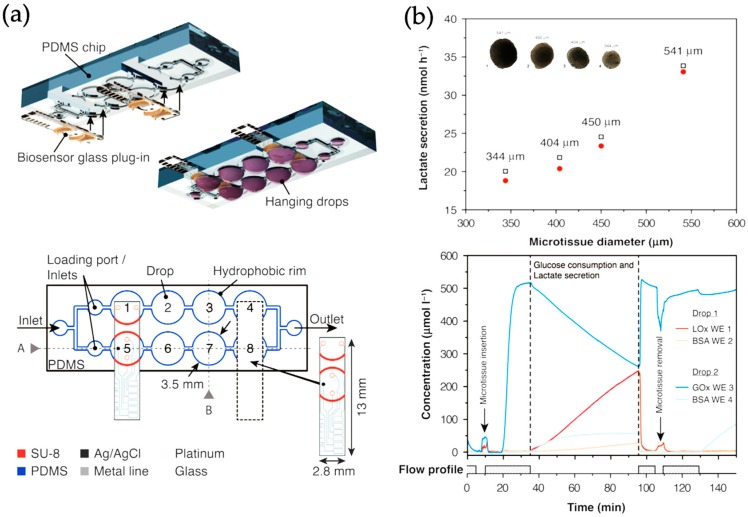
(**a**) Schematic view of hanging-drop chip for cancer organoids with an attached biosensor. (**b**) Measurement of glucose consumption and lactate secretion. (Adapted from [42] with permission from Nature Publishing Group 2019).

More multiplexed approaches in microtiter-plate format were reported by Misun et al. who adapted their well-established hanging drop spheroid multi-array by further implementing enzyme-based multi-analyte amperometric biosensors based on four electrode set-up (2xPt and 2xAg/AgCl) into a multiplexed cancer microtissue culture platform (see Figure 2) [42]. The integrated biosensors exhibited high sensitivity of 322 ± 41 nA mM^−1^ mm^−2^ for glucose and 443 ± 37 nA mM ^−1^ mm ^−2^ for lactate and demonstrated tissue-size-dependent, real-time detection of lactate secretion from human HCT116 colon cancer microtissues cultured in the hanging drops. Furthermore, glucose consumption and lactate secretion were monitored in parallel, and the impact of different culture conditions such as flow profiles and media compositions on cancer microtissue metabolism was recorded in real-time.

### 2.2. Monitoring in Endothelial & Epithelial Barrier-on-a-Chip Models

**Figure 3:**
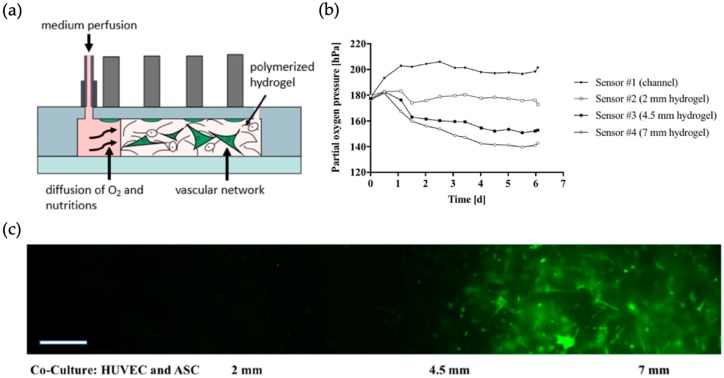
Microfluidic chip with 4 optical sensors for oxygen measurement in 3D vascular networks: (**a**) chip layout. (**b**) Simultaneous partial oxygen pressure measurement at 4 points in the chip. (**c**) Vascular network morphology (GFP-HUVEC cells are depicted green) at day 6 post-seeding with a medium perfusion speed of 5 μL/min. Scale bar 50 μm. (Reproduced from [29]).

From a simplistic viewpoint, the human body mainly consist of a variety of interconnected compartments, each separated by cellular barriers. Among these, tissue barriers between endothelial cells on one side and epithelial cells on the other side are of great interest, as they play a key role in transport between vasculature and organs, such as lung, skin, gastrointestinal tract etc. [43]. Understanding barrier function is key in drug screening efforts since biological barriers have to be considered as they affect drug uptake and transmission, bioaccumulation and therefore are key for successful drug development [44]. Another important aspect includes the determination of gas and nutrient gradients across tissue barriers, which are needed to recapitulate *in vivo* tissue conditions. For instance, Zirath et al. applied an opto-chemical sensing principle for investigation of 2D and 3D microfluidic cell models based on immobilized polystyrene beads pre-treated with an oxygen indicator solution (including a platinum-based dye (PtTPTBPF)) for non-invasive measurement of oxygen levels across the cultivation chamber (Figure 3). The authors showed how oxygen gradients within a cell-laden vascular barrier model based on a co-culture of human adipose-derived stem cells and HUVEC endothelial cells within a 3D fibrin scaffold can be tuned by selection of chip material as well as flow profiles [29].

**Figure 4:**
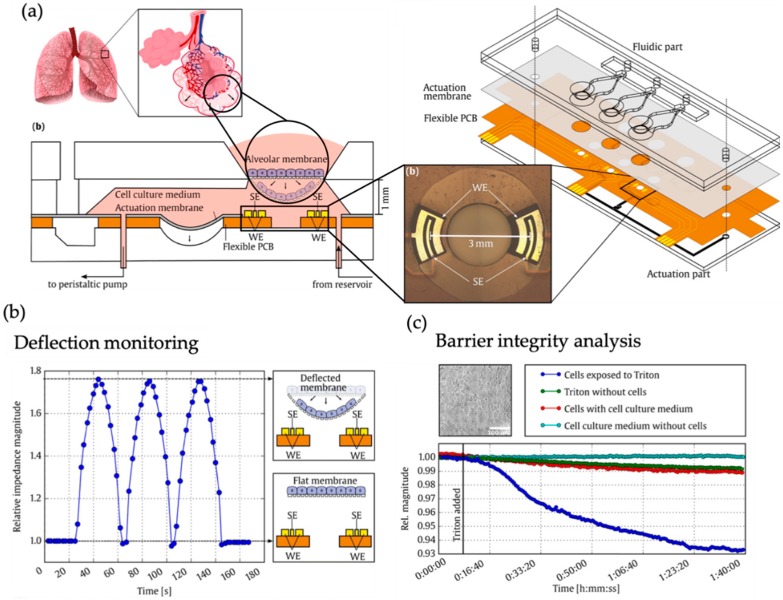
(**a**) Microimpedance tomography (MITO) system within lung-on-a-chip including sensing electrodes (SE) and working electrodes (WE). (**b**) Changes in impedance resulting from the respiratory movements of the cell culture membrane. The relative impedance changes result from the permeabilization of the epithelial monolayer. (**c**) Time-lapse relative impedance magnitude at a frequency of 1 kHz. (Adapted from [45] with permission from Elsevier).

In another report, Mermoud et al. employed a microimpedance tomography (MITO) sensing approach based on printed circuit board (PCB)-technology integrated in a flexible breathing lung-on-a-chip that impedance biosensors can be used for tracking of membrane deflection during breathing motion of the actuator membrane (Figure 4) [45]. The authors also demonstrated that due to the inherent sensitivity to the z-dimension of the electric field, relative impedance changes can also be linked to lung epithelial barrier break-down, thus cell membrane permeabilization can in principle be monitored at the alveolar membrane using the same biosensor.

**Figure 5:**
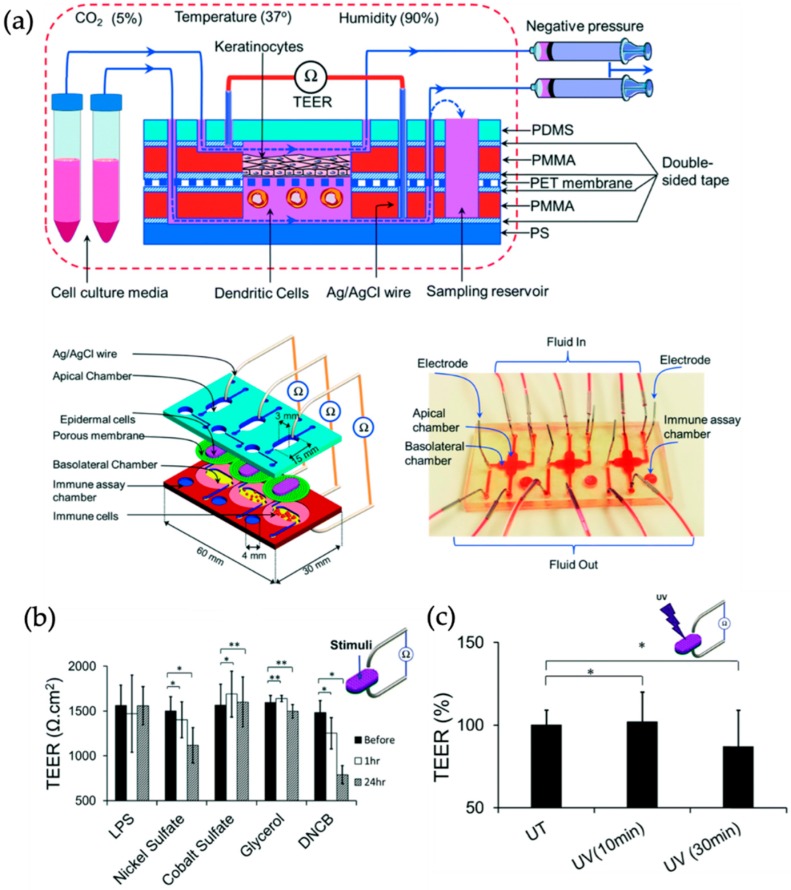
(**a**) Schematic cross-sectional sketch of the cell culture device with the perfusion setup as well as 3D schematic view of three parallel cell culture chambers including electrodes for TEER measurements and image of the fabricated chip. (**b**) TEER significantly decreases after 24 h of incubation with nickel sulfate while no obvious change is detectable after treatment with LPS, cobalt sulfate, or glycerol (* *p* < 0.05, ** *p* < 0.1). (**c**) The TEER value decreased to 82% of its original value upon UV irradiation. (Adapted from [47] with permission from The Royal Society of Chemistry).

Another example by Henry et al. used micro-structured electrodes manufactured on bottom and top polycarbonate chip layers that allow continuous and non-invasive measurements of epithelial barrier function on porous membranes based on industrial polymers [46]. Using this organ-on-a-chip platform the authors reported a gut and airway models allowing for Air-Liquid-Interface culture conditions and real-time readout to study transport of drugs across cellular barriers. Results of this study showed how intestinal epithelial barriers regenerate after exposure to 5 mM EGTA, a chelating agent that disrupts tight junctions and thus barrier integrity. Similarly, Ramadan and Ting reported a multi-layered membrane system integrated in a 3 × 1 array chip as human skin model using immune competent cells to advance allergic contact dermatitis and irritant contact dermatitis research. In brief, the immune competent *in vitro* skin model shown in Figure 5 is comprised of a 3D co-culture of immortalized human HaCat keratinocytes as epidermis barrier in combination with human leukemic U937 monocytes as human dendritic cell model. The dynamic perfusion of culture media significantly improved the tight junction formation measuring higher TEER values compared to static or air-liquid interface cultures up to 17 days. The inline TEER biosensor was also used to investigate the toxic effect of chemical stimulation as well as UV irradiation on the skin barrier integrity [47].

Another sensor-integrated biochip was reported by Shah et al. to investigate the gastrointestinal interface between human and microbes. Their approach incorporated three separated microchambers including medium perfusion, human epithelial cell (Caco-2) culture and microbial culture each equipped with inlet and outlet allowing individual control of physiochemical parameters and downstream analysis. Optical on-chip sensors were used for monitoring oxygen levels in real-time and transepithelial electrical resistance was measured to monitor cell growth and differentiation. Due to separated microchambers oxygen content in medium supply could be set individually and enabled establishment of *in vivo* like oxygen gradients. In a direct comparison to existing *in vivo* studies the authors were able to appropriately model the gastrointestinal human-microbe interface inside the microfluidic device [48]. A more engineering focused approach was presented by Pitsalidis et al. demonstrating in a recent proof-of-principle study how conducting polymers can not only be used as alternative electrode material but can also serve as electrochemical transistor configuration while simultaneously function as 3D cellular scaffold. The authors demonstrate how such an engineered tube-like system can be used to create a 3D cell culture compartment that can be used for barrier integrity studies of 2D kidney barriers under flow conditions [49].

### 2.3. Cardiac and Skeletal Muscle-on-a-Chip Systems with Integrated Biosensors

**Figure 6:**
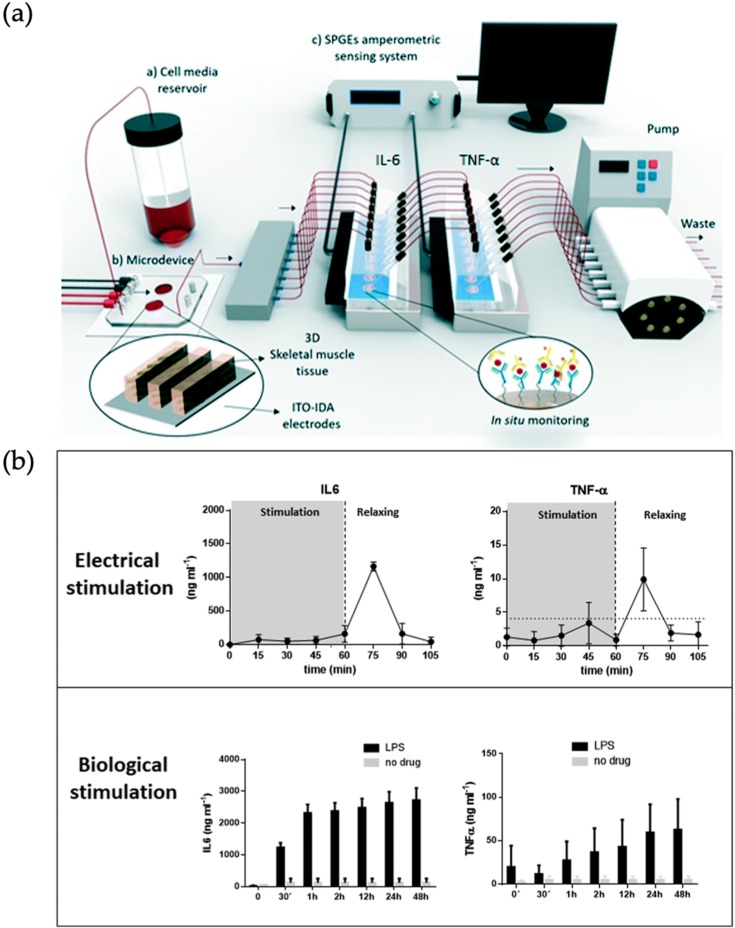
(**a**) Microfluidic setup for *in vitro* culture and stimulation of muscle tissue (murine C2C12 skeletal myoblasts) and subsequent analysis of IL-6 (Interleukin-6) and TNF-α (Tumor necrosis factor alpha) content. High-sensitivity screen-printed gold electrodes (SPGEs); indium tin oxide (ITO)- interdigitated array (IDA) electrodes (**b**) Increase in cytokine concentration was detected in the relaxation periods after electrical stimulation and also during stimulation with lipopolysaccharide (LPS). (Adapted from [50] with permission from The Royal Society of Chemistry).

Muscles in the human body can be characterized by their organotypic function into two types: skeletal muscle or cardiac muscle tissue. The latter is of special interest for drug screening studies to screen for unwanted side effects and conversion of drugs to cardiotoxic metabolites. Here, analysis of contractile forces and electrical signal propagation allow analysis of the muscle-on-a-chip models *in situ* [44]. Skeletal muscles-on-a-chip are mostly fabricated using cell-laden and differentiated hydrogel constructs, whereas cardiac microfluidic models can be either categorized as cell-laden hydrogel-based or embryoid body (EB)-derived cardiac bodies (CB). In turn, an *in vitro* model of skeletal muscles was embedded into a photopatterned polyacrylamide hydrogel-loaded microfluidic device by Agrawal et al. to evaluate muscle tissue morphogenesis, maturation, as well as drug cardiotoxicity. Using hydrogel anchoring pillars, the uniaxially-aligned, densely-packed cylindrical structure of native skeletal muscle was simulated. The deformation of anchoring pillars is used to determine the strain patterns of the embedded skeletal muscle cells in real time while they differentiate and form a multinucleated tissue bundle. Using finite element modeling prediction of toxin-induced changes in tissue architecture and passive muscle tension was demonstrated with cardiotoxin (CTX) exposure over several days [14]. Ortega et al. presented a microfluidic platform for electro-chemical online detection of myokine secretion after stimulation of skeletal muscle tissue *in vitro* (see Figure 6). In brief, murine C2C12 skeletal myoblasts were seeded within metacryliate-based GelMA-CMCMA hydrogels on chip and stimulated either electrically via indium tin oxide-interdigitated array electrodes or by a bacterial lipopolysaccharide solution. TNF-α and IL-6 concentrations were measured in an inline biosensor chamber array by antibody detection using functionalized gold electrodes. The measurement of these cytokines released by muscle tissue is envisioned to advance investigation of muscular disease onset and progression [50].

**Figure 7:**
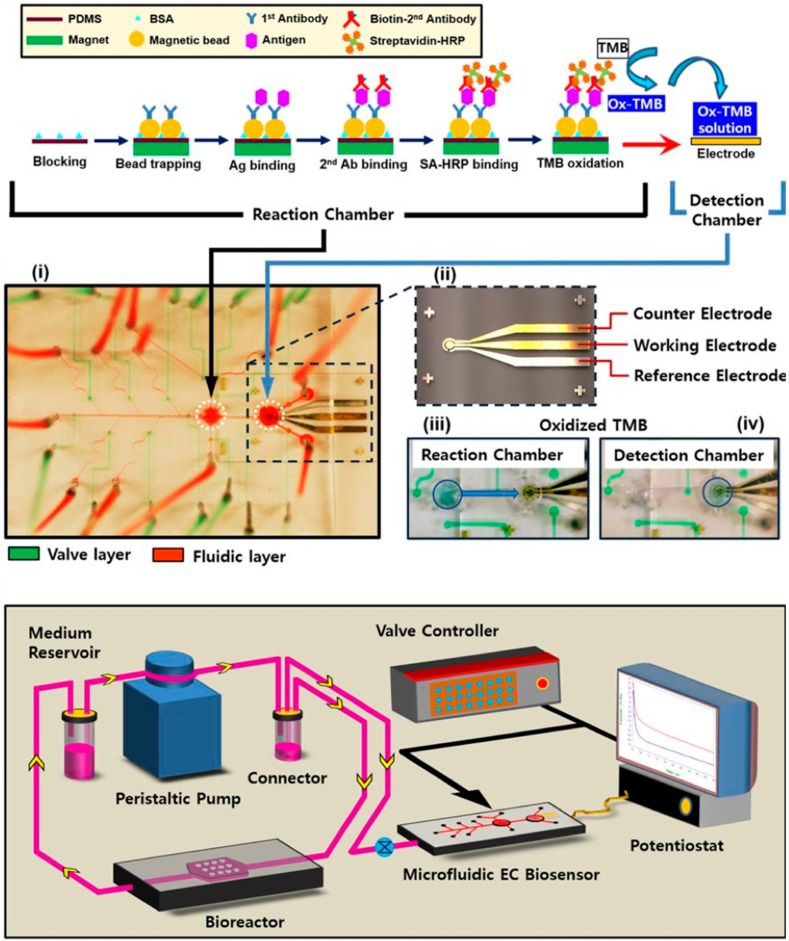
Immunosensing principle with the EC sensor for detection of target biomarkers and fabricated microfluidic sensing chip, (**i**) photograph (**ii**) microelectrodes, (**iii**) reaction chamber with oxidized TMB, (**iv**) transfer of oxidized TMB to the detection chamber and microfluidic sensing system. Bovine serum albumin (BSA); Horseradish peroxidase (HRP); Tetramethylbenzidine (TMB) (Reproduced from [57]).

Additionally, Huebsch et al. developed a heart-on-a-chip device for metabolically-driven maturation of human induced pluripotent stem cell derived cardiomyocytes (hiPSC-CM). By combining hiPSC-CM in a microfabricated PDMS device to build up a 3D cardiac microphysiological system (MPS) it was possible to improve immediate microtissue alignment and tissue specific extracellular matrix production. Through the 3D structure cells showed changes in behavior in regard to electrophysiology and pharmacology of MPS exposed to maturation media which wasn’t observed in 2D monolayers of the same cell type. Cell contraction was measured via PDMS micropillar deformation and linked to gene expression to observe the systematic combination of biophysical stimuli and metabolic cues to improve the electrophysiological maturation of hiPSC-derived cardiomyocytes [51]. Furthermore, Caluori et al. have combined an electrochemical multielectrode array (MEA) with AFM measurements to record the beating rate of cardiomyocyte organoids as well as the deformation of the cardiac cluster during contraction [52]. This setup not only allows to couple electrical and mechanical measurements, but by synchronizing the acquisition also the delay between electrical activity and mechanical reaction can be measured. Cardiac bodies were either differentiated from human pluripotent stem cell line CCTL14 or an induced dystrophin-deficient cell line reprogrammed from fibroblasts of a patient affected by Duchenne Muscular Dystrophy (DMD). The β-adrenergic stimulation by isoproterenol and antagonist verapamil addressed ionotropic and chronotropic cell line-dependent differences and for the first time, a distinctive beating-force relation for DMD was measured in a 3D *in vitro* organ-on-a-chip model. Instead of using conventional gold electrodes which are usually fabricated using photolithographic methods, Inácio et al. [53] reported on PEDOT:PSS polymer electrodes, which can be easily ink-jet printed using a material printer onto many different substrates such as glass, silicon or polymers. Instead of small area electrodes, which are commonly used on multi-electrode arrays, the authors used large area electrodes to record the beating rate from whole embryoid bodies with enhanced signal quality. Another benefit of PEDOT:PSS electrodes is the reduced thermal noise as the large interfacial capacitance decreases, because of the lower polymer cell culture media interface [53]. Also, PEDOT:PSS electrodes can be printed onto flexible and conformal substrates with improved performance that show lower noise and increased impedance in contrast to conventional MEAs with gold or titanium nitride electrodes as reported by Koutsouras et al. [54] Shin et al. presented single-use as well as upgraded multi-use electrochemical biosensors for time-resolved monitoring of secreted cardiac and hepatic biomarkers. [55] For automation of the microfluidic bead-based electrochemical immunosensor microvalves were integrated on-chip to allow for programmable operations of the immunoassay including immobilization of biorecognition elements, antigen binding, washing, and electrochemical sensing. Doxorubicin induced increase in cardiac creatine kinase (CK-MB) levels in organoids were monitored inline using the presented automated and miniaturized electrochemical biosensing approach for heart-on-a-chip models (see Figure 7) [56,57].

### 2.4. In Situ Analysis of Human Microfluidic Nervous Systems and Blood Brain Barrier Models

**Figure 8:**
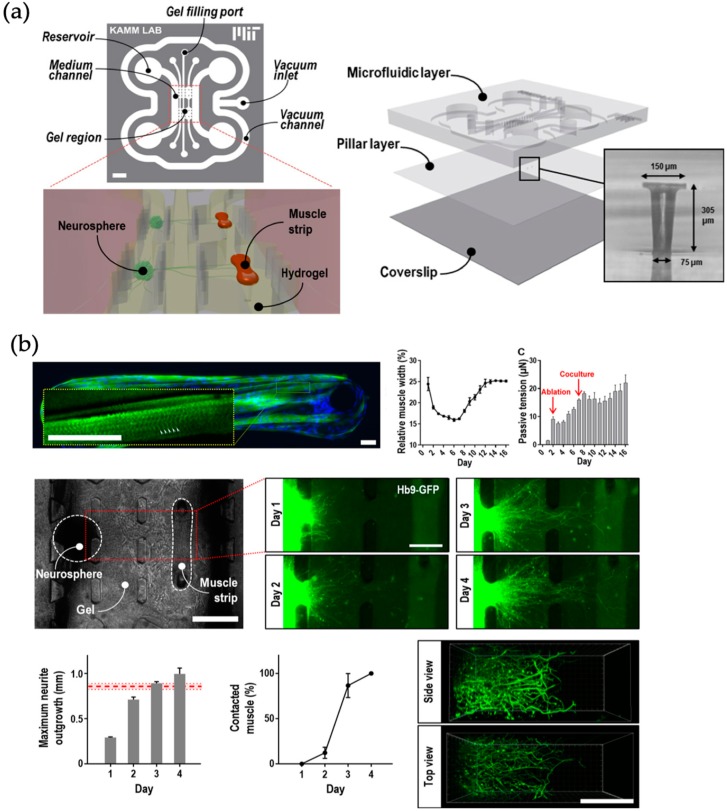
(**a**) Microfluidic device temporarily bonded by vacuum and composed of two layers: (I) Microfluidic layer and (II) PDMS membrane featuring two sets of two capped pillars measuring muscle deflection. (**b**) Muscle and neurite growth over 16 days and muscle contraction measurement. (Reprinted from [61] with permission from AAAS).

The nervous system is the central routing organization for various types of signals in the human body and relays information from the brain to the rest of the body and vice versa. Closely connected to this system is the blood brain barrier, a functional membrane separating the blood stream from brain interstitial fluid yet still allow signal transmission [58]. Animal models for these structures have shown their limitations and therefore *in vitro* (microfluidic) models have been increasingly used in recent years [59]. These include disease models for Parkinson’s and Alzheimer’s disease [60], the two most common neurodegenerative disorders, and the ability to transfer compounds through the blood brain barrier is of great interest for drug development concerning these diseases. For instance, a microfluidic device for the formation of optically excitable, 3D, compartmentalized motor units (neuro-muscular interfaces) was reported by Uzel et al. (see Figure 8). This *in vitro* platform enhanced the physiology of a motor unit by 3D co-culturing and compart-mentalization of mouse embryonic stem cell derived motor neurons and skeletal muscle cells within an extra cellular matrix scaffold. Passive force transducers (e.g., PDMS pillars deformation) are used for quantification of the muscle contraction similarly to previously published reports by Huebsch et al. The quantification is used to gain a better understanding culture of functional differentiated motor neurons and myofibers, the observation of 3D axonal outgrowth with the hydrogel, and the formation of functional neuromuscular junctions. The platform is envisioned to be used for drug screening to understand the influence of different substances on the muscle contraction by a defined optical stimulus of the neuronal cell structure [61].

**Figure 9:**
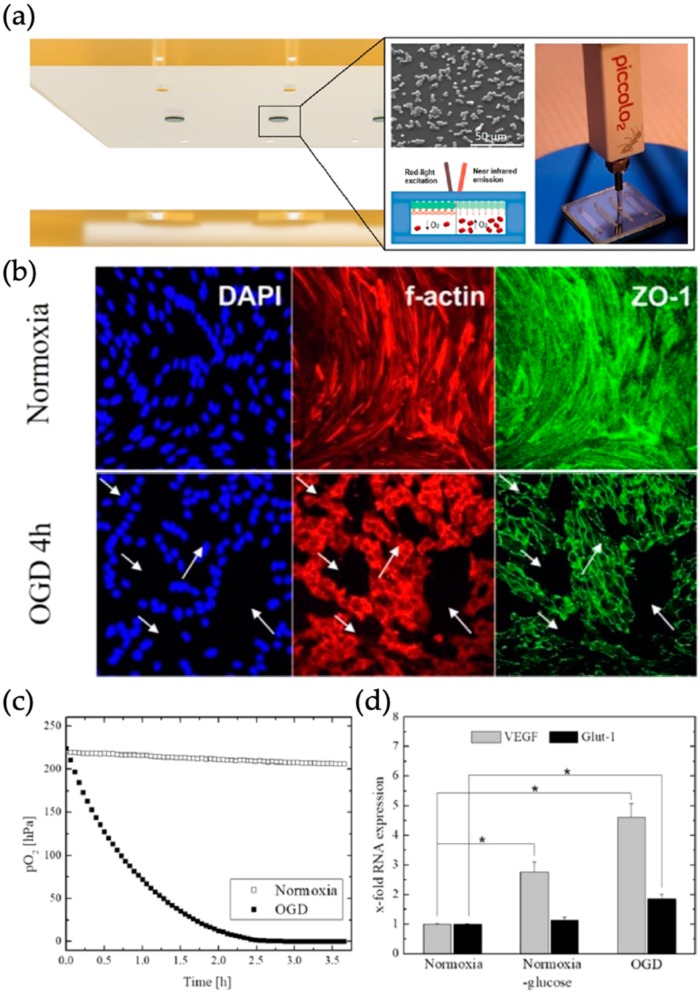
(**a**) Overview of microfluidic chip and oxygen sensing principle, particles have a size of 5 μm. (**b**) Morphology changes after 4-h period of normoxia conditions and under oxygen-glucose-deprivation (OGD). Arrows indicate ruptures in the cell barrier. (**c**) Oxygen measurement during normoxia conditions and under OGD. (**d**) Expression of VEGF and GLUT-1 in blood-brain-barrier model under normoxia (with and without glucose) and under OGD conditions. Reproduced with permission from [62] Copyright 2019 American Chemical Society.

Alternatively, Sticker et al. introduced a microfluidic stroke-on-a-chip model with an oxygen scavenging biochip material in combination with integrated opto-chemical oxygen sensing microbeads, which can be used to recreate desired reduced oxygen concentrations mimicking *in vivo* conditions during stroke (see Figure 9). Precise control over oxygen levels was achieved by variations in microfluidic layout, flow rate and material curing protocols. Biomedical relevance of the chip material was demonstrated by showing a disease model consisting of blood brain barrier cerebEND cells for ischemic stroke. Cytoskeletal morphology changes indicating this disruption, due to oxygen-glucose deprivation (OGD) were presented in combination with the decreased partial oxygen-pressure and upregulation of vascular endothelial growth factor (VEGF) and glucose transporter GLUT-1 [62].

## 3. Bio Multi Organ and Human-on-a-Chip Systems

**Figure 10:**
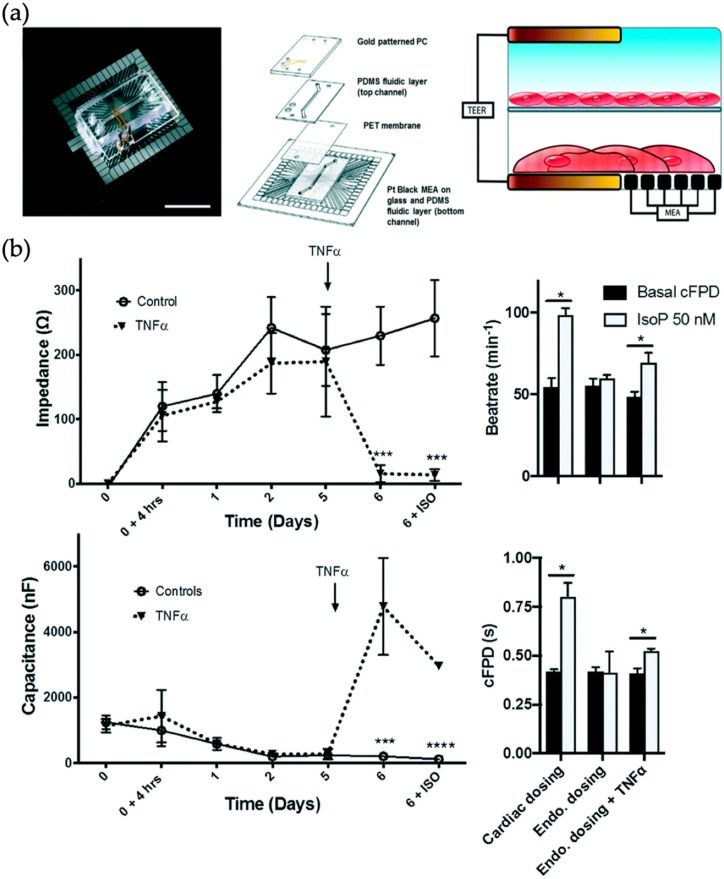
(**a**) TEER–MEA chip—endothelial cell layer on top of the PET membrane and cardiomyocytes on top of MEA—measuring TEER of both cell layers. (**b**) Influence of TNF-α on TEER and capacitance of the endothelial cell layer. (Adapted from [65] with permission from The Royal Society of Chemistry).

Multi-organs-on-a-chip systems advance single organ systems by enabling the analysis of organ-organ interactions as well as investigating ADME (absorption, distribution, metabolism and elimination) processes for drug evaluation. Especially in pharmaceutical studies early identification of interactions between different tissues and organs is of high interest to avoid waste of time and monetary assets. Despite this interest in multi-organs-on-a-chip systems there is still room for improvement, particularly in long-term testing and automation [63]. As an example, Maoz et al. presented a combinatorial approach joining TEER biosensors with a Multi-Electrode array (so-called “TEER-MEA”) to measure the influence of cardiac targeted drugs like Isoproterenol across inflamed endothelial cell barriers with higher vascular permeability (see Figure 10) [64]. The multi-layered device comprised of two culture chambers separated via a porous membrane. The cardiomyocytes were grown on a multi electrode array which forms the lower chamber. The top TEER Electrode was integrated into the cover of the upper chamber, while the bottom TEER electrode was integrated next to the Multi-Electrode Array chip. By adding isoproterenol to the top chamber and challenging the epithelial layer with tumor necrosis factor alpha (TNF-α) the microdevice recorded an increase of beating rate of the cardiomyocytes due to the presence of inflammatory cytokines.

**Figure 11:**
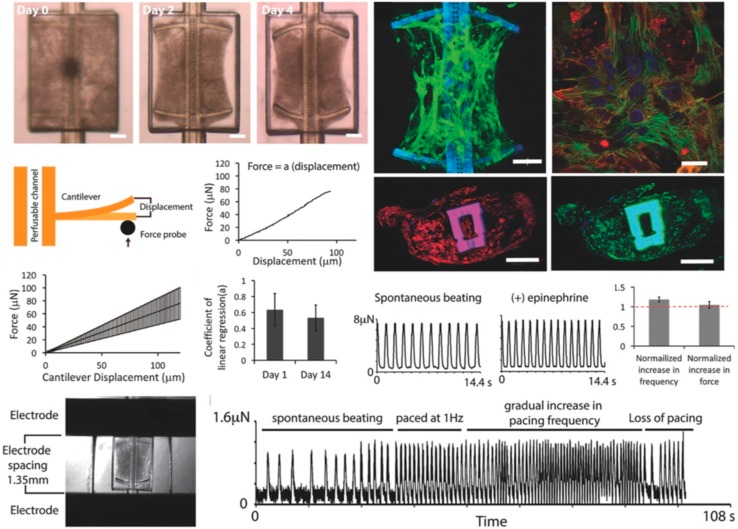
Heart model with integrated cantilever for continuous electrical measurement of cardiomyocyte activity. (Reproduced from [65] with permission from John Wiley & Sons, Inc., Hoboken, USA).

A more complex and multiplexed vascular model was reported by Lai et al. shown in Figure 11 combining printed 3D organ structures with built-in perfusable vascular structures for investigating cancer metastasis. A combined blood vessel, liver, heart and solid tumor model in a 96-well plate structure was taken to study cancer metastasis by non-invasive analysis of permeability, metabolism, biomechanics and migration. For biosensing of cardiac tissue compartment flexible cantilever-based read-out of cardiac tissue contraction was incorporated into the micro structures and beta-adrenergic agonist, epinephrine, perfused through the internal vasculature, lead to an immediate increase in tissue contraction frequency [65]. In addition, cancer cell invasion assays as well as drug screening applications were demonstrated using this microtiter plate sized platform.

**Figure 12:**
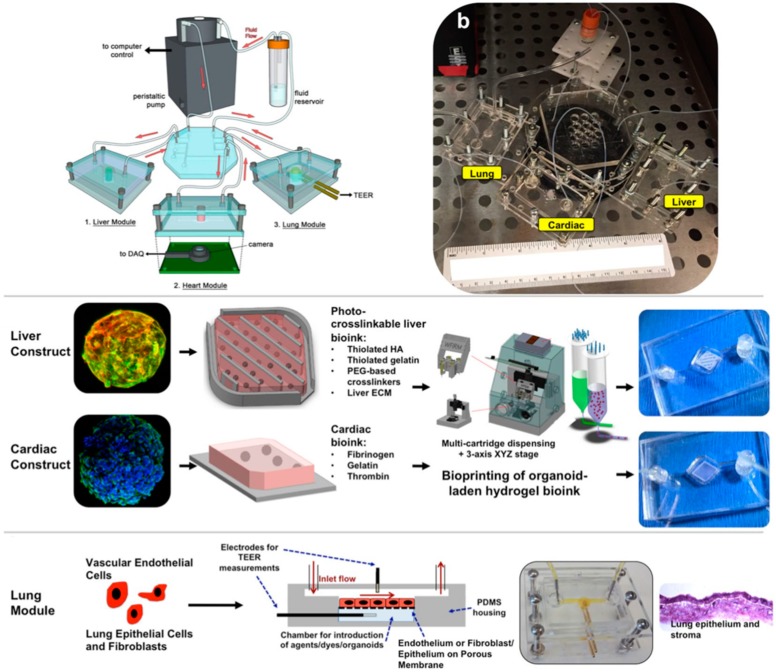
“plug-and-play” 3-tissue-representative organ-on-a-chip system. Liver and cardiac modules are created by bioprinting spherical organoids within customized bioinks, resulting in 3D hydrogel constructs that are placed into the microreactor devices. Lung modules are formed by creating layers of cells over porous membranes within microfluidic devices. Introduction of TEER sensors allows monitoring of tissue barrier function integrity over time. (Reproduced from [68]).

Another approach reported by Zhang et al. describes a multi-sensor organ-on-a-chip platform for online measurements based on multiple sensors along the heart–liver axis. This modular platform was used to investigate organoid behavior on-chip and included monitoring capabilities for environmental parameters like pH, oxygen and temperature. Environmental sensors were based on light absorption due to phenol red (pH), quenching effects of an oxygen sensitive ruthenium dye and a simple probe for temperature measurement. Organoid investigation was performed by miniature microscopes and label-free electrochemical immunosensing, which was achieved by electrodes that could be functionalized and regenerated. High adaptability and relevance of their system was shown as it was not only capable of running fully automated for at least 5 days but could also be used for investigation of drug induced (e.g., acetaminophen) organ toxicity in healthy and cancer heart (derived from iPSC-CMs) and liver (derived from primary hepatocytes or HepG2/C3A hepatocellular carcinoma cells) models [66]. To study the effects of the hepatic metabolism on off-target cardiotoxicity Oleaga et al. built a gravity driven microfluidic device with incorporated multielectrode and cantilever arrays to study primary aspects of the *in vivo* crosstalk between heart and liver and for pharmacological studies. The system was used to screen cardiotoxicity induced by drugs and their metabolites produced primarily from hepatic cytochrome P450 (CYP) metabolism. Two different drugs were applied for the validation of the system: cyclophosphamide and terfenadine. Cyclophosphamide is a non-cardiotoxic parent drug which that metabolite upon liver generates a cardiotoxic substance, whereas terfenadine is a cardiotoxic parent drug which leads to a non-cardiotoxic metabolite after hepatic metabolism. Those interconnections of each drug can be observed within the two-organ-device and quantified through cantilever read out of the beating cardiac tissue [67]. Skardal et al. advanced the heart and liver organ-on-a-chip system by implementing a lung model in the same system (see Figure 12). The individual organ models were bioprinted and consisted of primary human hepatocytes, stellate cells and Kupffer cells for liver organoids, induced pluripotent stem cells for heart organoids and lung fibroblasts, epithelial and endothelial cells for lung membranes. Analysis of the system was performed by measurement of cardiac beat rates by real-time imaging, antibody-binding by impedance change and barrier function monitoring by trans endothelial electrical resistance (as an addition to traditional supernatant analysis). When investigating potentially toxic effects of bleomycin on the lung organ model, adverse effects on the cardiac organoid was observed, which did not occur when treating the cardiac model alone with the same compound. This indicates the importance of multi-organ models to reveal interactions between multiple tissues and organs [68].

**Figure 13:**
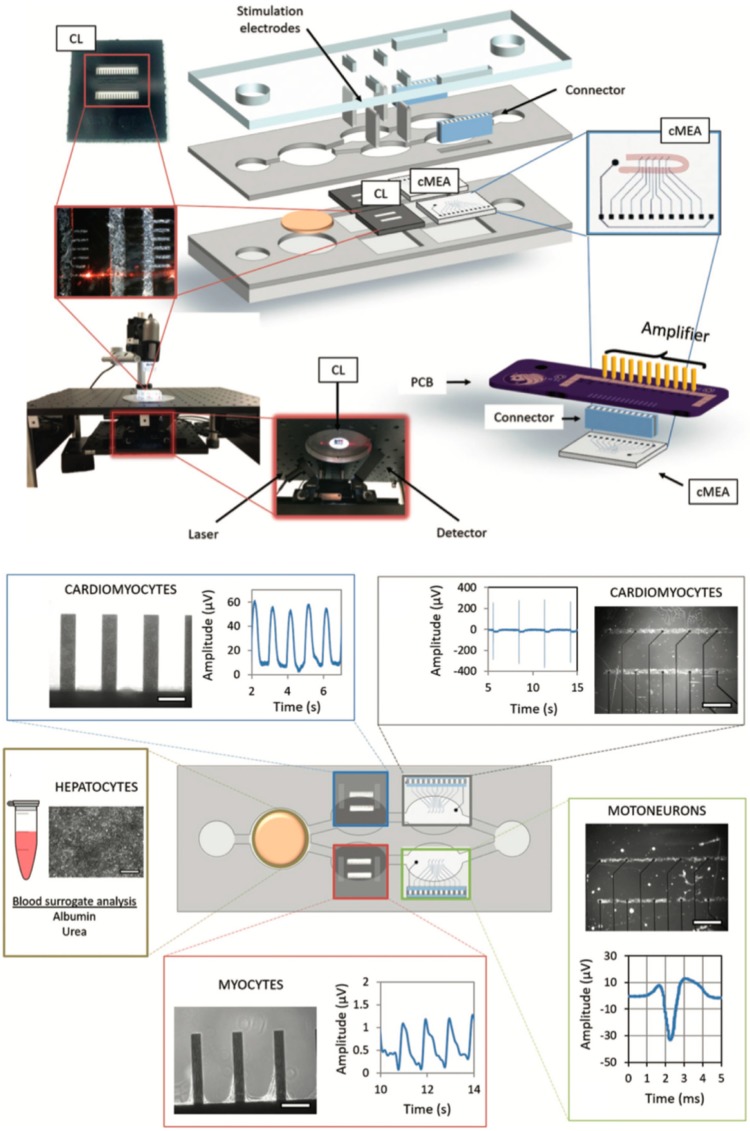
Non-invasive monitoring of cellular function in a 4-organ system measuring the mechanical and electrical functional activity of cardiomyocytes and motoneurons as well as secretion of hepatocytes. (Reproduced from [69]).

Next, Oleaga et al. reported an advanced and functional human serum-free four-organ platform including cardiac, muscle, neuronal and liver modules to evaluate multi-organ toxicity of five drugs under continuous flow conditions utilizing a pumpless platform for two weeks (see Figure 13) [69].

The pharmacological relevance was evaluated for five drugs with known side effects after a 48 h drug treatment all drug treatments were in general agreement with published toxicity results from human and animal data for doxorubicin, atorvastatin, acetaminophen, N-acetyl-m-aminophenol as well as valproic acid. Nonetheless, aside from the impressive off-chip analysis schemes only a single biosensor based on silicon cantilever technology was integrated for spontaneous or induced cardiomyocyte contraction force measurements as a function of deflection of the microfabricated cantilevers. This obvious lack in continuous monitoring was improved 2018 by integration of a non-invasive electrical functional multielectrode array (MEA)-based readout strategy for the motoneuron and cardiomyocyte compartments [70]. Cultivation time of heart, liver, skeletal muscle, and nervous system modules was also improved to 28 days in serum-free conditions again using a pumpless reservoir tilting system, which allows the monitoring of cellular function for chronic toxicity studies in a two-sensor body-on-a-chip system.

In contrast to normally fixed microfluidic designs for multi-organs-on-a-chip and body-on-a-chip systems, modular microfluidic platforms have been introduced over the years to increase the potential application of multi-organ chips by creating more flexible plug & play designs. Recently Gaio et al. present a modular “Cytostretch” platform with membrane variants including porous, stretchable micro-electrode array-integrated, micro-patterned and titanium thin-film strain membranes. The authors showed how state-of-the-art silicon-based micro-fabrication techniques allows generation of a versatile yet easily scalable toolkit for organ-on-a-chip devices and show very rudimentary proof-of-principle studies on immune cell chemotaxis, field potential/impedance recordings of human induced pluripotent stem cell (hiPSCs) and more physiological phenotype of hiPSC-derived cardiomyocytes on patterned membranes compared to flat membrane. In a more recent effort, the same authors presented protocols on how to mold and assemble these modular “cytostrech” modules in a 2 × 2 array suitable for multi-organ- or body-on-a-chip applications [71].

## 4. Conclusions

Here, the latest progress of biosensing strategies integrated within organs-on-a-chip models were reviewed including microphysiological models for (i) individual as well as (ii) interconnected multi-organs-on-a-chip (“Body/human-on-a-chip”) as summarized in Table 1. Integration of biosensors is a promising strategy to enhance the impact of organ-on-a-chip technology for drug screening especially when single functional organ units are interconnected to become a body-/human-on-a-chip system, where the monitoring of individual organ function may require a combination of different sensing principles (e.g., barrier integrity of an endothelial or epithelial compartment, beating frequency of a muscle/cardiac model, tumor metabolism and/or cytokine release of a target tissue upon inflammatory stimulation). Interestingly, electrochemical and optical biosensors are the only types of biosensors that are used nowadays for organs-on-a-chip systems including analysis of barrier integrity (TEER), neuronal activity (MEAs), cytokine release (electrochemical cytokine biosensors) and/or optical biosensors based on deflection of flexible structure (Pillar deformation/cantilever deflection). It has to be noted here that there is a huge palette of other than the already mentioned bio- and microsensors ready to be integrated within organ- and body-on-a-chip devices. This technological gap cannot be simply explained by lack of expertise and/or interdisciplinarity since even commercially available sensor types based on field-effect transistors or nanowires are still to be integrated in the near future. Among organs that can be tested using such biosensors obviously cardiac and skeletal muscle-on-a-chip are predominant type of organs monitored using cantilever-based deflection readout for muscle contraction or spontaneous beating of cardiac bodies. The other predominant biosensor application involves human *in vitro* barriers including skin, intestine, BBB, vasculature, lung and kidney which can be tested non-invasively *in situ* using tetra- or bipolar electrochemical biosensor (TEER, impedance). These types of biosensors are well established in trans-well technology and familiar to cell biologists. Additionally, these microsensors can be easily integrated into microfluidic biochips using either lift-off of shadow masking metal thin-film deposition. This means that they can easily replace invasive fluorescent dye leakage assays, where the fluorescent probe can interfere with the actual molecule/drug transport. To our surprise, no recent studies were reported on the application of MEA technology for 3D neuronal organ-on-a-chip models, which could be interesting to probe neuronal activity not only for disease models such as Parkinson’s or Alzheimers but can be used also for neurotoxicological screening of novel therapeutic strategies. In particular, multiplexed electrochemical biosensors can be used to monitor either neuronal activity using MEAs complementary to calcium imaging using fluorescent probes (e.g., FURA-2, etc.) Also, neurotransmitter release can be potentially monitored *in situ* using state-of-the-art amperometry on-chip in the vicinity of the 3D neuronal model to reduce analyte oxidation, which is a frequent challenge in neurotransmitter research. The next decade of research on establishment of organs-on-a-chip systems needs to focus on how to actually probe and monitor relevant key organ functions, which is even more challenging when considering multi-organs-on-a-chip and body-on-a-chip devices, where each tissue and organ type may demand a specific biosensing strategy. Here, the current state-of-the-art heavily relies on off-chip analysis and live-cell microscopy techniques with fluorescently labelled/transfected cell lines. The reason behind this off-chip analysis trend can be simply answered as follows: (i) off-chip analysis schemes are often already established protocols used in most bio-labs worldwide (e.g., PCR, ELISA, Colorimetric assays, FACS, Histology etc.); (ii) implementation and integration of bio- and microsensors demands more specialized and expensive infrastructure in comparison to microfluidic technology that is rather cheap; (iii) the more interdisciplinary the organ-on-a-chip approach the harder it is to find employees that can operate these complex microsystems; (iv) The majority of researchers in life sciences can operate a fluorescent microscope. Fortunately, again the palette of established bio- and microsensors is huge and recently very promising new types of sensors are published that could potentially be integrated within organ-and body-on-a-chip system to make the read-out of human physiology *in vitro* more accurate, reliable and scalable. The overall challenge is to motivate engineers and biologists to leave the comfort zone and give in to the high interdisciplinarity of sensor-integrated organs- and body-on-a-chip systems.

## Figures and Tables

**Table 1:** Overview of organs-on-a-chip and microphysiological systems with integrated biosensors.

Organ	Simulated Organ Function	Cell Type (+Primary, -Cancer, * Stem Cell)	Tissue Architecture	Sensing Principle	References
Skeletal muscle	Tissue morphogenesis and maturation and effects to cardiotoxins	C2C12 mouse murine myoblast (-)	3D cell-laden hydrogel structures	Pillar deformation (fluorescence microscopy) and finite element method	[14]
Vascular network	Oxygen gradients in vascular networks	HUVEC (human umbilical vein endothelial cells) (+) and ASC (adipose-derived stem cells) (+)	3D cell-laden hydrogel structures	Oxygen sensing by fluorescence measurements of oxygen sensitive platinum-based dye (PtTPTBPF)	[29]
Pancreatic islets	Glucose concentration-dependent micro-organ activity	Β-cells of pancreatic islets adult male C57BL/6 mice (+)	3D islets	Electrical activity sensing of pancreatic islets by multielectrode array	[40]
Liver	Mitochondrial respiration	HepG2/C3A (-)	3D cell-laden hydrogel structures	Oxygen sensing by phosphorescence of a ruthenium dye and glucose and lactate sensing by oxidation of platinum electrodes	[41]
Cancer (colon) microtissue	Glucose and lactate metabolism	Fluorescent human colon carcinoma cell line HCT116 eGFP (-)	3D spheroid	Glucose and lactate sensing by using electrodes functionalized with oxidase enzymes and amperometry	[42]
Lung	Mechanical strain of alveolar barrier during breathing	Human type II alveolar epithelial-like A549 cells (-)	Barrier model	Barrier movement and membrane permeabilization sensing by real-time measurement of resistivity changes in three impedimetric coplanar electrodes.	[45]
Lung and gut	Barrier function formation (by stem cell differentiation)	Primary human airway epithelial cells (hAECs) (+) and human Caco2 intestinal epithelial cells (-)	Barrier model	Barrier integrity sensing by TEER measurements	[46]
Skin	Allergic und irritant contact dermatitis	Immortalized human keratinocytes (HaCaT) (-) and human leukemic monocyte lymphoma cell line (U937) (-)	Barrier model	Barrier integrity and tight junction formation sensing by TEER measurements	[47]
Gastrointestinal human-microbe interface	Transcriptional, metabolic and immunological response	Caco-2 (-), CCD-18Co (+) and CD4+T cells (+)	Barrier model	Optodes for oxygen sensing and TEER measurements for cell growth and differentiation	[48]
Kidney	Barrier function	Canine epithelial kidney cells (MDCKII) (-) and human telomerase-immortalized fibroblasts (-)	3D barrier model	Barrier integrity sensing by transconductance measurements	[49]
Skeletal muscle	Myokine secretion	Murine C2C12 skeletal myoblasts (-)	3D cell-laden hydrogel structures	Myokine concentration measurement by functionalized gold electrodes	[50]
Heart	Formation of 3D cardiac microphysiological system	Human induced pluripotent stem cell derived cardiomyocytes (hiPSC-CMs) (*)	3D cell-laden PDMS structure	Cardiac cell contraction sensing by micropillar deformation	[51]
Heart	Cardiac beat rate	Human embryoid stem cell line CCTL14 (*) and human induced pluripotent stem cells (*)	3D organoid	Cardiomyocyte beating force sensing by multielectrode array and atomic force microscopy measurements	[52]
Embryoid body (cardiac cells)	Autonomous beat rate of embryoid bodies	Mouse embryonic stem cells (mESC) differentiated cardiomyocytes (*)	3D embryoid body	Cardiac beat rate sensing by voltage and displacement current measurement by large area electrodes	[53]
Pancreatic Islets	Electrical Activity of single cells and islets	Pancreatic islets of mice and human (+)	3D islets	Action potential local field potential measurement by multielectrode array	[54]
Heart	Cardiac biomarker secretion	Human embryonic stem cell-derived cardiomyocytes (ESC-CMs) (*)	3D cell-laden hydrogel structures	Creatine kinase (CK)-MB sensing by impedance measurements using an aptamer functionalized microelectrode	[55]
Liver	Hepatic biomarker secretion	Human primary hepatocytes (+)	3D cell-laden hydrogel structures	Biomarker sensing by impedance measurements regeneratable gold electrodes	[56]
Liver	Hepatic biomarker secretion	HepG2 (-) and primary human hepatocytes (+)	3D cell-laden hydrogel structures	Immobilization of recognition molecules by magnetic microbeads and subsequent electrochemical measurement	[57]
Skeletal muscle and lower motor neurons	Neuromuscular junction	Mouse embryonic stem cell (mESC) line HBG3 (Hb9-GFP) (*) and mouse myoblasts C2C12 (-)	3D cell-laden hydrogel structures	Muscle contraction sensing by passive force transducers (pillar deformation)	[61]
Blood brain barrier	Disease model of ischemic stroke	Murine brain endothelial cells (cerebEND) (-)	Barrier model	Oxygen sensing by fluorescence measurements of palladium-based dyes (PdTPTBFP)	[62]
Heart	Barrier function and electrical activity of endothelialized myocardium	Human umbilical cord vascular endothelial cells (HUVECs) (+) and human induced pluripotent stem cell-derived cardiomyocytes (iPSC-CMs) (*)	Barrier model	Barrier integrity and electrical activity sensing by TEER-multielectrode array measurements	[64]
Blood vessel, heart, liver	Cancer metastasis	Human umbilical vein endothelial cells (HUVEC) and human hepatocellular carcinoma (HepG2) (-) and human cardiomyocytes differentiation of human pluripotent stem cell (hPSC) line BJ1D (*)	3D cell-laden hydrogel structures	Cardiac beat frequency sensing by fluorescence microscopy and computational analysis of microcantilevers.	[65]
Heart and liver	Organ toxicity	Human induced pluripotent stem cell-derived cardiomyocytes (iPSC-CMs) (*), primary hepatocytes (+) and HepG2/C3A hepatocellular carcinoma cells (-)	3D organoids	pH sensing by light absorption of phenol red, oxygen sensing by fluorescence measurements of quenching effects of oxygen sensitive ruthenium dye and immunosensing by functionable electrodes	[66]
Heart and liver	Cardiotoxicity (primarily from hepatic cytochrome P450 (CYP) metabolism)	Human induced pluripotent stem cell (iPSc) derived cardiomyocytes (*) and human primary hepatocytes (+)	2D monolayers	Multielectrode array for electrical activity sensing and cantilever array for sensing of cardiac mechanical function	[67]
Heart, liver and lung	Organ toxicity	Hepatic stellate cells (HSCs) (+), primary human hepatocytes (+), Kupffer cells (+), induced pluripotent stem cell-derived cardiomyocytes (iPSC CMs) (*), human primary cardiac fibroblasts (+), lung microvasculature endothelial cells (+), airway stromal mesenchymal cells (+), bronchial epithelial cells (+)	3D organoids	Cardiac beat rate measurement by real-time imaging and computational analysis, antibody-binding by impedance measurement and barrier function by TEER measurement	[68]
Heart, liver, skeletal muscle and neuronal network	Organ toxicity	Human hepatocellular carcinoma HepG2/C3A (-), human induced pluripotent stem cell (iPSc) differentiated cardiomyocytes (*), human skeletal myofibers (+), human motoneurons differentiated from human spinal cord stem cell line (hSCSC) (*) and human iPSc differentiated cortical-like neurons (*)	2D monolayers	Cardiomyocyte contraction (force) sensing by cantilever deflection (laser beam reflection) (69, 70) and electrical activity of cardiomyocytes or motoneurons by a multielectrode array (70)	[69,70]
Heart	Immune cell chemotaxis, stretching characteristics	Human induced pluripotent stem cells (hiPSCs) (*) and human induced pluripotent stem cell (hiPSCs)-derived cardiomyocytes (*)	2D monolayers	Electrical field potential sensing of cardiomyocytes under membrane stretch by multielectrode array and membrane stretching sensing by measurement of electrical resistance change in strain gauges	[71]

## References

[1] Mak I.W., Evaniew N., Ghert M. (2014). Lost in translation: Animal models and clinical trials in cancer treatment. Am. J. Transl. Res..

[2] Liu K.D., Humphreys B.D., Endre Z.H. (2017). The ten barriers for translation of animal data on AKI to the clinical setting. Intensive Care Med..

[3] Leenaars C.H., Kouwenaar C., Stafleu F.R., Bleich A., Ritskes-Hoitinga M., De Vries R.B., Meijboom F.L. (2019). Animal to human translation: A systematic scoping review of reported concordance rates. J. Transl. Med..

[4] Rothbauer M., Rosser J.M., Zirath H., Ertl P. (2019). Tomorrow today: Organ-on-a-chip advances towards clinically relevant pharmaceutical and medical *in vitro* models. Curr. Opin. Biotechnol..

[5] Huh D., Matthews B.D., Mammoto A., Montoya-Zavala M., Hsin H.Y., Ingber D.E. (2010). Reconstituting organ-level lung functions on a chip. Science.

[6] van Midwoud P.M., Verpoorte E., Groothuis G.M. (2011). Microfluidic devices for *in vitro* studies on liver drug metabolism and toxicity. Integr. Biol..

[7] Lee J., Choi J.-H., Kim H.J. (2016). Human Gut-on-a-Chip Technology: Will this Revolutionize Our Understanding of IBD and Future Treatments.

[8] Weber E.J., Chapron A., Chapron B.D., Voellinger J.L., Lidberg K.A., Yeung C.K., Wang Z., Yamaura Y., Hailey D.W., Neumann T. (2016). Development of a microphysiological model of human kidney proximal tubule function. Kidney Int..

[9] Wang Y.I., Abaci H.E., Shuler M.L. (2017). Microfluidic blood–brain barrier model provides *in vivo*-like barrier properties for drug permeability screening. Biotechnol. Bioeng..

[10] Günther A., Yasotharan S., Vagaon A., Lochovsky C., Pinto S., Yang J., Lau C., Voigtlaender-Bolz J., Bolz S.-S. (2010). A microfluidic platform for probing small artery structure and function. Lab Chip.

[11] Bachmann B., Spitz S., Rothbauer M., Jordan C., Purtscher M., Zirath H., Schuller P., Eilenberger C., Ali S.F., Mühleder S. (2018). Engineering of 3D pre-vascular networks within fibrin hydrogel constructs by microfluidic control over reciprocal cell signaling. Biomicrofluidics.

[12] Jastrzebska E., Tomecka E., Jesion I. (2016). Heart-on-a-chip based on stem cell biology. Biosens. Bioelectron..

[13] Marsano A., Conficconi C., Lemme M., Occhetta P., Gaudiello E., Votta E., Cerino G., Redaelli A., Rasponi M. (2016). Beating heart on a chip: A novel microfluidic platform to generate functional 3D cardiac microtissues. Lab Chip.

[14] Agrawal G., Aung A., Varghese S. (2017). Skeletal muscle-on-a-chip: An *in vitro* model to evaluate tissue formation and injury. Lab Chip.

[15] Lee J.S., Romero R., Han Y.M., Kim H.C., Kim C.J., Hong J.-S., Huh D. (2016). Placenta-on-a-chip: A novel platform to study the biology of the human placenta. J. Matern.-Fetal Neonatal Med..

[16] Johnson B.N., Lancaster K.Z., Hogue I.B., Meng F., Kong Y.L., Enquist L.W., McAlpine M.C. (2016). 3D printed nervous system on a chip. Lab Chip.

[17] Arık Y.B., van der Helm M.W., Odijk M., Segerink L.I., Passier R., van den Berg A., van der Meer A.D. (2018). Barriers-on-chips: Measurement of barrier function of tissues in organs-on-chips. Biomicrofluidics.

[18] Wu J., Chen Q., Liu W., He Z., Lin J.-M. (2017). Recent advances in microfluidic 3D cellular scaffolds for drug assays. TrAC Trends Anal. Chem..

[19] Kaushik G., Leijten J., Khademhosseini A. (2017). Concise review: Organ engineering: Design, technology, and integration. Stem Cells.

[20] Wall M., Butler D., El Haj A., Bodle J.C., Loboa E.G., Banes A.J. (2018). Key developments that impacted the field of mechanobiology and mechanotransduction. J. Orthop. Res..

[21] Wang X., Phan D.T., Sobrino A., George S.C., Hughes C.C., Lee A.P. (2016). Engineering anastomosis between living capillary networks and endothelial cell-lined microfluidic channels. Lab Chip.

[22] Ertl P., Sticker D., Charwat V., Kasper C., Lepperdinger G. (2014). Lab-on-a-chip technologies for stem cell analysis. Trends Biotechnol..

[23] Sticker D., Lechner S., Jungreuthmayer C., Zanghellini J., Ertl P. (2017). Microfluidic migration and wound healing assay based on mechanically induced injuries of defined and highly reproducible areas. Anal. Chem..

[24] Shen C., Meng Q., Zhang G. (2013). Increased curvature of hollow fiber membranes could up-regulate differential functions of renal tubular cell layers. Biotechnol. Bioeng..

[25] Raghavan V., Rbaibi Y., Pastor-Soler N.M., Carattino M.D., Weisz O.A. (2014). Shear stress-dependent regulation of apical endocytosis in renal proximal tubule cells mediated by primary cilia. Proc. Natl. Acad. Sci. USA.

[26] Ergir E.E., Bachmann B., Redl H.R., Forte G., Ertl P. (2018). Small force, big impact: Next generation organ-on-a-chip systems incorporating biomechanical cues. Front. Physiol..

[27] Rothbauer M., Praisler I., Docter D., Stauber R., Ertl P. (2015). Microfluidic impedimetric cell regeneration assay to monitor the enhanced cytotoxic effect of nanomaterial perfusion. Biosensors.

[28] Kim H.J., Huh D., Hamilton G., Ingber D.E. (2012). Human gut-on-a-chip inhabited by microbial flora that experiences intestinal peristalsis-like motions and flow. Lab Chip.

[29] Zirath H., Rothbauer M., Spitz S., Bachmann B., Jordan C., Müller B., Ehgartner J., Priglinger E., Mühleder S., Redl H. (2018). Every breath you take: Non-invasive real-time oxygen biosensing in two-and 3D microfluidic cell models. Front. Physiol..

[30] Novak R., Ranu N., Mathies R.A. (2013). Rapid fabrication of nickel molds for prototyping embossed plastic microfluidic devices. Lab Chip.

[31] Tsao C.-W. (2016). Polymer microfluidics: Simple, low-cost fabrication process bridging academic lab research to commercialized production. Micromachines.

[32] Lafleur J.P., Joensson A., Senkbeil S., Kutter J.P. (2016). Recent advances in lab-on-a-chip for biosensing applications. Biosens. Bioelectron..

[33] Gabardo C., Soleymani L. (2016). Deposition, patterning, and utility of conductive materials for the rapid prototyping of chemical and bioanalytical devices. Analyst.

[34] Huh D., Hamilton G.A., Ingber D.E. (2011). From 3D cell culture to organs-on-chips. Trends Cell Biol..

[35] Rothbauer M., Wartmann D., Charwat V., Ertl P. (2015). Recent advances and future applications of microfluidic live-cell microarrays. Biotechnol. Adv..

[36] Gruber P., Marques M.P., Szita N., Mayr T. (2017). Integration and application of optical chemical sensors in microbioreactors. Lab Chip.

[37] Pires N., Dong T., Hanke U., Hoivik N. (2014). Recent developments in optical detection technologies in lab-on-a-chip devices for biosensing applications. Sensors.

[38] Kieninger J., Weltin A., Flamm H., Urban G.A. (2018). Microsensor systems for cell metabolism–from 2D culture to organ-on-chip. Lab Chip.

[39] Caballero D., Kaushik S., Correlo V., Oliveira J.M., Reis R., Kundu S. (2017). Organ-on-chip models of cancer metastasis for future personalized medicine: From chip to the patient. Biomaterials.

[40] Perrier R., Pirog A., Jaffredo M., Gaitan J., Catargi B., Renaud S., Raoux M., Lang J. (2018). Bioelectronic organ-based sensor for microfluidic real-time analysis of the demand in insulin. Biosens. Bioelectron..

[41] Bavli D., Prill S., Ezra E., Levy G., Cohen M., Vinken M., Vanfleteren J., Jaeger M., Nahmias Y. (2016). Real-time monitoring of metabolic function in liver-on-chip microdevices tracks the dynamics of mitochondrial dysfunction. Proc. Natl. Acad. Sci. USA.

[42] Misun P.M., Rothe J., Schmid Y.R.F., Hierlemann A., Frey O. (2016). Multi-analyte biosensor interface for real-time monitoring of 3D microtissue spheroids in hanging-drop networks. Microsyst. Nanoeng..

[43] Sakolish C.M., Esch M.B., Hickman J.J., Shuler M.L., Mahler G.J. (2016). Modeling barrier tissues *in vitro*: Methods, achievements, and challenges. EBioMedicine.

[44] Wang Y.I., Oleaga C., Long C.J., Esch M.B., McAleer C.W., Miller P.G., Hickman J.J., Shuler M.L. (2017). Self-contained, low-cost Body-on-a-Chip systems for drug development. Exp. Biol. Med..

[45] Mermoud Y., Felder M., Stucki J., Stucki A., Guenat O. (2017). Microimpedance tomography system to monitor cell activity and membrane movements in a breathing lung-on-chip. Sens. Actuators B Chem..

[46] Henry O.Y.F., Villenave R., Cronce M.J., Leineweber W.D., Benz M.A., Ingber D.E. (2017). Organs-on-chips with integrated electrodes for trans-epithelial electrical resistance (TEER) measurements of human epithelial barrier function. Lab Chip.

[47] Ramadan Q., Ting F.C. (2016). *In vitro* micro-physiological immune-competent model of the human skin. Lab Chip.

[48] Shah P., Fritz J.V., Glaab E., Desai M.S., Greenhalgh K., Frachet A., Niegowska M., Estes M., Jäger C., Seguin-Devaux C. (2016). A microfluidics-based *in vitro* model of the gastrointestinal human–microbe interface. Nat. Commun..

[49] Pitsalidis C., Ferro M.P., Iandolo D., Tzounis L., Inal S., Owens R.M. (2018). Transistor in a tube: A route to 3D bioelectronics. Sci. Adv..

[50] Machuca M.A.O., Garibay X.G.F., Castaño A.G., De Chiara F., Albors A.H., Trias J.B., Azcon J.R. (2019). Muscle-on-a-chip with on-site multiplexed biosensing system for *in situ*-monitoring of secreted IL-6 and TNF-α. Lab Chip.

[51] Huebsch N., Charrez B., Siemons B., Boggess S.C., Wall S., Charwat V., Jæger K.H., Montiel F.T.L., Jeffreys N.C., Deveshwar N. (2018). Metabolically-driven maturation of hiPSC-cell derived heart-on-a-chip. bioRxiv.

[52] Caluori G., Pribyl J., Pesl M., Jelinkova S., Rotrekl V., Skladal P., Raiteri R. (2019). Non-invasive electromechanical cell-based biosensors for improved investigation of 3D cardiac models. Biosens. Bioelectron..

[53] Inácio P., Mestre A., de Medeiros M., Asgarifar S., Canudo J., Elamine Y., Santos J., Morgado J., Braganca J., Biscarini F. (2017). Bioelectrical Signal Detection Using Conducting Polymer Electrodes and the Displacement Current Method. IEEE Sens. J..

[54] Koutsouras D.A., Perrier R., Villarroel Marquez A., Pirog A., Pedraza E., Cloutet E., Renaud S., Raoux M., Malliaras G.G., Lang J. (2017). Simultaneous monitoring of single cell and of micro-organ activity by PEDOT:PSS covered multi-electrode arrays. Mater. Sci. Eng. C.

[55] Shin S.R., Zhang Y.S., Kim D.-J., Manbohi A., Avci H., Silvestri A., Aleman J., Hu N., Kilic T., Keung W. (2016). Aptamer-Based Microfluidic Electrochemical Biosensor for Monitoring Cell-Secreted Trace Cardiac Biomarkers. Anal. Chem..

[56] Shin S.R., Kilic T., Zhang Y.S., Avci H., Hu N., Kim D., Branco C., Aleman J., Massa S., Silvestri A. (2017). Label-Free and Regenerative Electrochemical Microfluidic Biosensors for Continual Monitoring of Cell Secretomes. Adv. Sci..

[57] Riahi R., Shaegh S.A.M., Ghaderi M., Zhang Y.S., Shin S.R., Aleman J., Massa S., Kim D., Dokmeci M.R., Khademhosseini A. (2016). Automated microfluidic platform of bead-based electrochemical immunosensor integrated with bioreactor for continual monitoring of cell secreted biomarkers. Sci. Rep..

[58] van Der Helm M.W., Van Der Meer A.D., Eijkel J.C., van den Berg A., Segerink L.I. (2016). Microfluidic organ-on-chip technology for blood-brain barrier research. Tissue Barriers.

[59] Yi Y., Park J., Lim J., Lee C.J., Lee S.-H. (2015). Central nervous system and its disease models on a chip. Trends Biotechnol..

[60] Ahadian S., Civitarese R., Bannerman D., Mohammadi M.H., Lu R., Wang E., Davenport-Huyer L., Lai B., Zhang B., Zhao Y. (2018). Organ-on-a-chip platforms: A convergence of advanced materials, cells, and microscale technologies. Adv. Healthc. Mater..

[61] Uzel S.G., Platt R.J., Subramanian V., Pearl T.M., Rowlands C.J., Chan V., Boyer L.A., So P.T., Kamm R.D. (2016). Microfluidic device for the formation of optically excitable, 3D, compartmentalized motor units. Sci. Adv..

[62] Sticker D., Rothbauer M., Ehgartner J., Steininger C., Liske O., Liska R., Neuhaus W., Mayr T., Haraldsson T., Kutter J.P. (2019). Oxygen Management at the Microscale: A Functional Biochip Material with Long-Lasting and Tunable Oxygen Scavenging Properties for Cell Culture Applications. ACS Appl. Mater. Interfaces.

[63] Zhao Y., Kankala R.K., Wang S.-B., Chen A.-Z. (2019). Multi-organs-on-chips: Towards long-term biomedical investigations. Molecules.

[64] Maoz B.M., Herland A., Henry O.Y.F., Leineweber W.D., Yadid M., Doyle J., Mannix R., Kujala V.J., FitzGerald E.A., Parker K.K. (2017). Organs-on-Chips with combined multi-electrode array and transepithelial electrical resistance measurement capabilities. Lab Chip.

[65] Lai B.F.L., Huyer L.D., Lu R.X.Z., Drecun S., Radisic M., Zhang B. (2017). InVADE: Integrated Vasculature for Assessing Dynamic Events. Adv. Funct. Mater..

[66] Zhang Y.S., Aleman J., Shin S.R., Kilic T., Kim D., Shaegh S.A.M., Massa S., Riahi R., Chae S., Hu N. (2017). Multisensor-integrated organs-on-chips platform for automated and continual *in situ* monitoring of organoid behaviors. Proc. Natl. Acad. Sci. USA.

[67] Oleaga C., Riu A., Rothemund S., Lavado A., McAleer C.W., Long C.J., Persaud K., Narasimhan N.S., Tran M., Roles J. (2018). Investigation of the effect of hepatic metabolism on off-target cardiotoxicity in a multi-organ human-on-a-chip system. Biomaterials.

[68] Skardal A., Murphy S.V., Devarasetty M., Mead I., Kang H.-W., Seol Y.-J., Zhang Y.S., Shin S.-R., Zhao L., Aleman J. (2017). Multi-tissue interactions in an integrated three-tissue organ-on-a-chip platform. Sci. Rep..

[69] Oleaga C., Bernabini C., Smith A.S.T., Srinivasan B., Jackson M., McLamb W., Platt V., Bridges R., Cai Y., Santhanam N. (2016). Multi-Organ toxicity demonstration in a functional human *in vitro* system composed of four organs. Sci. Rep..

[70] Oleaga C., Lavado A., Riu A., Rothemund S., Carmona-Moran C.A., Persaud K., Yurko A., Lear J., Narasimhan N.S., Long C.J. (2018). Long-Term Electrical and Mechanical Function Monitoring of a Human-on-a-Chip System. Adv. Funct. Mater..

[71] Gaio N., Waafi A., Vlaming M.L.H., Boschman E., Dijkstra P., Nacken P., Braam S.R., Boucsein C., Sarro P.M., Dekker R. A multiwell plate Organ-on-Chip (OOC) device for in-vitro cell culture stimulation and monitoring. Proceedings of the 2018 IEEE Micro Electro Mechanical Systems (MEMS).

